# Shannon entropy approach reveals relevant genes in Alzheimer’s disease

**DOI:** 10.1371/journal.pone.0226190

**Published:** 2019-12-31

**Authors:** Alfonso Monaco, Nicola Amoroso, Loredana Bellantuono, Eufemia Lella, Angela Lombardi, Anna Monda, Andrea Tateo, Roberto Bellotti, Sabina Tangaro

**Affiliations:** 1 Istituto Nazionale di Fisica Nucleare (INFN), Sezione di Bari, Bari, Italy; 2 Department of Physics ‘Michelangelo Merlin’, University of Bari ‘Aldo Moro’, Bari, Italy; University of Texas Health Science Center at San Antonio, UNITED STATES

## Abstract

Alzheimer’s disease (AD) is the most common type of dementia and affects millions of people worldwide. Since complex diseases are often the result of combinations of gene interactions, microarray data and gene co-expression analysis can provide tools for addressing complexity. Our study aimed to find groups of interacting genes that are relevant in the development of AD. In this perspective, we implemented a method proposed in a previous work to detect gene communities linked to AD. Our strategy combined co-expression network analysis with the study of Shannon entropy of the betweenness. We analyzed the publicly available GSE1297 dataset, achieved from the GEO database in NCBI, containing hippocampal gene expression of 9 control and 22 AD human subjects. Co-expressed genes were clustered into different communities. Two communities of interest (composed by 72 and 39 genes) were found by calculating the correlation coefficient between communities and clinical features. The detected communities resulted stable, replicated on two independent datasets and mostly enriched in pathways closely associated with neuro-degenative diseases. A comparison between our findings and other module detection techniques showed that the detected communities were more related to AD phenotype. Lastly, the hub genes within the two communities of interest were identified by means of a centrality analysis and a bootstrap procedure. The communities of the hub genes presented even stronger correlation with clinical features. These findings and further explorations on the detected genes could shed light on the genetic aspects related with physiological aspects of Alzheimer’s disease.

## Introduction

Alzheimer’s disease (AD) is the most common type of dementia in aging population (up to the 70% of dementia’s cases) [1]. The World Alzheimer Report 2016 affirmed that 47 million people are affected by dementia and it is expected that over 131 million people will develop dementia by 2050 [2]. Pathological processes are involved in AD such as intraneuronal formation of NeuroFibrillary Tangles (NFTs) [3, 4], abnormal *β*-amyloid production [5–8], extracellular deposition of senile plaques, early loss of synapses [9], oxidative stress [10, 11], and inflammation [8, 12–14]. To date, the pathogenesis of AD remains largely unknown and there is no cure for this disease, but treatment can still help in reducing symptoms and providing a better quality of life [15]. Converging evidence suggests that complex diseases result from association of several interacting genes possibly merging in molecular process. This suggests the existence of genetic communities that may be relevant for AD. Hence, recognition of the genetic basis of the disease is absolutely required to understand the biology of AD and to discover novel pharmacological treatments. In particular, the study of specific gene communities could facilitate the identification of therapeutic targets or candidate biomarkers.

In this work, we investigated gene expression data from a “publicly accessible microarray database obtained from AD and control human hippocampus” to detect gene communities relevant for AD. In this context, we implemented a gene co-expression network analysis addressed to formalize and integrate information related to multiple genes. In this approach, we modeled data through a network whose edges model the correlation between gene expressions and genes are the nodes of the graph [16]. Several methods have been proposed to investigate gene co-expression networks [17–19]. For example Weighted Gene Co-expression Network Analysis (WGCNA) has become widely adopted to provide a network identification based on the correlation of gene expression of a microarray database [20]. In particular, WGCNA can be used for identifying clusters of co-expressed genes with highly correlated expression (communities). In this work we implemented a hard threshold analysis and a community detection method, proposed in a previous work [21] and used in an international competition [22], based on the study of information content of the network. In the original paper [21] we employed this procedure on gene expression data linked to schizophrenia, now we applied the method to AD data. We identified 127 gene communities; two of them resulted significantly correlated with Mini Mental State Examination (MMSE) and NFT value. We verified that the two communities are stable and mostly replicated on two independent datasets. We also compared the proposed method, in addition to WGCNA, to three traditional clustering methods and three network reconstruction techniques. We performed a gene set enrichment analysis and a study to identify the hub genes of the two communities of interest applying centrality metrics and a bootstrap procedure. The communities of the found hub genes appear highly correlated with AD phenotype, and they could represent targets for a future AD therapy.

## 1 Materials and methods

**Fig 1:**
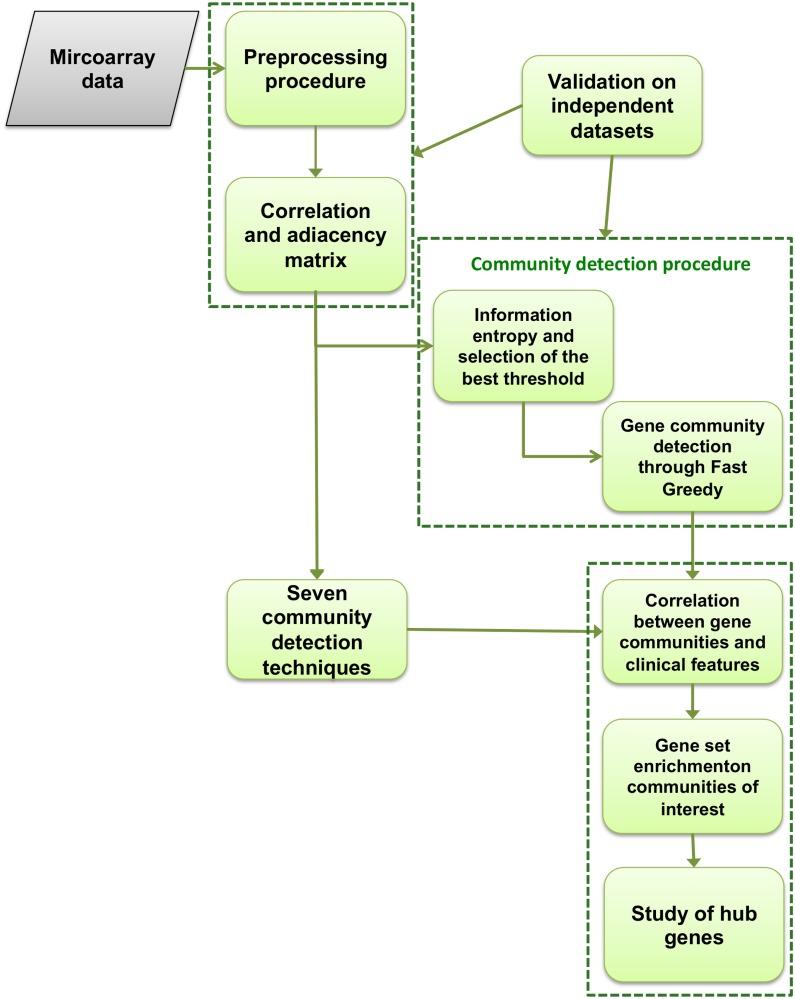
Flowchart of the methodology. After a preprocessing analysis, we implemented a community detection procedure based on hard threshold analysis and information theory proposed in a previous work [21]. We conduced a correlation analysis between gene communities and MMSE value and NFT score. We repeated the analysis through different community detection techniques and we proposed a comparison with our method. Subsequently a gene set enrichment analysis and a hub gene study have been conduced. At last, we validated our procedure on two independent datasets.

In this work we analyzed data achieved from the GEO database in NCBI (Gene Expression Omnibus, https://www.ncbi.nlm.nih.gov/geo/). The data entry number of database is GSE1297. This dataset was developed from the work of Blalock [14] and contains hippocampal gene expression of 31 human subjects: 9 control and 22 AD. Clinical data as the severity of the disease, NFT value, Braak stage, MMSE score, sex, age, and Post-Mortem Interval (PMI) values are also included in the GSE1297 dataset. Data analysis procedure is summarized in Fig 1 and it consists of five main steps:

1. firstly, the normalized data was downloaded and a preprocessing analysis was implemented to combine expression values of multiple probes for one gene and to select genes with larger standard deviations. Secondly, correlation measures were used to construct the co-expressed gene network;
2. a hard threshold analysis was performed to compute the best threshold value, by using an information theory-based approach. Once the best threshold has been chosen, a community detection method was performed;
3. Person’s correlation analysis between gene communities and clinical features (MMSE, NFT) was performed and communities of interest were identified; gene set enrichment analysis on communities of interest was applied and the hub genes of these modules were identified;
4. on the co-expressed gene network built in the step 1, community identification procedures by means of seven module detection algorithms were applied. Hence, correlation between gene communities and clinical features (MMSE, NFT) was computed. A comparison with results of our method was proposed;
5. steps 1 and 2 of the pipeline was repeated on two independent datasets to validate the proposed community detection method.

In the point 5 of the pipeline we analyzed hippocampal data of two other databases of GEO: GSE48350 and GSE29378. Both datasets contains gene expression of 38 and 16 control, 18 and 17 AD human subjects respectively.

### 1.1 Preprocessing data analysis

We downloaded the normalized data containing expression values of 22, 283 probes. The probes without corresponding annotation information were removed. In case of multiple probes for the same gene, probe with high Median Absolute Deviation (MAD) values was retained for further analysis. We chose MAD because it is a measure of dispersion, robust to outliers [23]. After the previous filtering, the standard deviation of the remaining gene expressions was calculated and values were sorted in decreasing order. Finally, we applied a selection criterion on the standard deviation to get a compromise between maximizing the information contained in gene expression data and minimizing the number of genes.

### 1.2 Correlation measures for a network of co-expressed genes

The network of co-expressed genes was built considering gene selected through the preprocessing procedure as nodes and analyzing their expressions for *N* = 31 subjects under investigation. In particular, given genes *i* and *j*, with expressions on the cohort {*i*_*a*_}_*a* = 1,…,*N*_ and {*j*_*b*_}_*b* = 1,…,*N*_ respectively, we computed the absolute value [24]:

$$\begin{array}{c} d_{ij}=\left|r_{ij}\right| \end{array}$$ (1)

where *r*_*ij*_ is the Pearson’s pairwise correlation:

$$\begin{array}{c} r_{ij}=\frac{\sum_{a=1}^{N}\left(i_{a}-\bar{i}\right)\sum_{b=1}^{N}\left(j_{b}-\bar{j}\right)}{\sqrt{\sum_{a=1}^{N}{\left(i_{a}-\bar{i}\right)}^{2}}\sqrt{\sum_{b=1}^{N}{\left(j_{b}-\bar{j}\right)}^{2}}} \end{array}$$ (2)

with $\bar{i}$ and $\bar{j}$ mean values of the two expression distributions. The result is adjacency matrix *C*, in which each elements *c*_*ij*_ = *f*(*d*_*ij*_) is a function of the correlation between expressions of genes *i* and *j* and characterizes the strength of the corresponding link in the network. Usually, two different thresholding methods to elaborate this matrix are considered: the soft and the hard thresholding. In the latter approach, the elements of *C* are defined as:

$$\begin{array}{c} c_{ij}=signum\left(d_{ij},th\right)=\{\begin{array}{cc} 1 & \text{if}\;|d_{ij}|\geq th\\ 0 & \text{if}\;|d_{ij}|<th \end{array} \end{array}$$ (3)

where *th* is the selected threshold value. In this work, links were introduced in the network of co-expressed genes by means of a hard thresholding procedure [21], and the optimal threshold values was selected through information theory.

### 1.3 Information entropy based on betweenness to select the best threshold value

Betweenness *b*_*i*_ is a network centrality measure that evaluates the role of a node in connecting other pairs of nodes. For a complex network with *M* nodes the betweenness of the node *i* is defined as:

$$\begin{array}{c} b_{i}=\sum_{j,k,j\neq k}^{M}\frac{n_{jk}\left(i\right)}{n_{jk}} \end{array}$$ (4)

where *n*_*jk*_ indicates the number of geodesics between node *j* and *k*, and *n*_*jk*_(*i*) is the number of geodesics between the same genes, passing through node *i*. A geodesic between two nodes *j* and *k* is defined as the shortest path connecting them. Analyzing Eq 4 it is evident that betweenness has a crucial importance for graph characterization [25–27].

We implemented the method described in [21] and used in an international competition [22] to select the best threshold value with a hard threshold procedure based on information entropy [28] of the co-expression network betweenness. For a complex network with M nodes we defined the entropy based on betweenness as:

$$\begin{array}{c} H_{bet}=-\sum_{i=1}^{M}b_{i}log_{2}\left[b_{i}\right] \end{array}$$ (5)

where *b*_*i*_ is the betweenness of the *i*-*th* node defined by Eq 4. Since a system with maximum entropy value represents a system with maximum information content [29], we computed the entropy based on betweenness for the network of co-expressed genes, varying threshold values, and we chose the threshold value that maximized Eq 5.

### 1.4 Community detection through Fast Greedy algorithm

Once the best threshold was fixed, we applied the Fast Greedy [30] algorithm to split the whole network in communities. Fast Greedy is based on greedy optimization and it characterizes the community structure through the modularity. Modularity is based on the number of intra-community and inter-community links [31–35] and it allows the comparison of different partitions of the network. The modularity of a given partition is represented by the number of edges falling within groups minus the expected number in an equivalent random network. In particular we used the Fast Greedy algorithm described in [36]. Briefly, this method optimizes the modularity using three data structures:

- the matrix of modularity variation Δ*Q*_*ij*_ between communities *i* and *j*;
- a max-heap *H* composed of the largest element of each row of Δ*Q*_*ij*_ and of the labels, related to the communities *i* and *j*;
- an ordinary vector array which contains the sums of the elements of each row of the matrix *e*_*ij*_. This matrix is the fraction of edges that joins vertices in community *i* to vertices in community *j* [36].

The use of max-heap *H*, which organizes the data in the form of binary trees, allows to update the matrix *e*_*ij*_ faster than the Newman’s algorithm reported in [32]. In the present work, through an iterative procedure, we only selected communities that contained at least 3 and a maximum of 100 genes. Thus we referred to the concept of community, which was used in other international community detection studies as the DREAM challenge [22]. We implemented the hard threshold procedure described in the previous section, first on the whole network and then on all communities with more than 100 genes. As a matter of fact, communities comprising hundreds of genes are often too populated to gain meaningful biological insights [37].

### 1.5 Correlation analysis of gene modules with clinical phenotype

**Fig 2:**
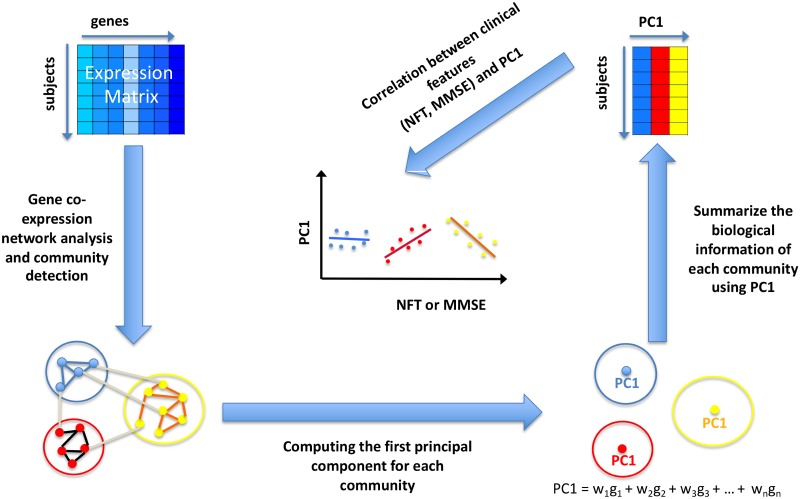
Schematic view of the correlation procedure between module eigengene and clinical features. After the community detection procedure, we computed the module eigengene (first principal component, PC1, of the community) for each found community. Then we implemented a Pearson’s correlation analysis between PC1 and clinical features (MMSE and NFT).

The association, between the communities found in the step 2 of the pipeline and clinical phenotype, was investigated by means of Pearson’s correlation analysis. In particular, we computed the correlation of the module eigengene (first principal component, PC1, of the community) of each community with AD clinical features (MMSE, NFT). In other words, we synthetized the biological information of each community in one eigengene using principal component analysis [38–40]. Fig 2 shows a schematic view of the correlation procedure implemented.

To verify the robustness of significant correlations we implemented a procedure in which random subsets of 10 genes are removed from the communities of interest before to compute the first principal component. We repeated this procedure 100 times and for each sampling we calculated correlations with the clinical features through the method described in this section.

### 1.6 Gene set enrichment analysis

Only for the communities significantly correlated to clinical phenotype, we performed a gene set enrichment analysis using GSEA [41] web-tool. Through this tool we evaluated the overlap of the communities found in the step 3 of the pipeline, with the Molecular Signatures Database (MSigDB) [42]. Hence, we computed an estimate of the statistical significance to highlight common processes, pathways, and underlying biological themes. The overlap was measured by means of the hypergeometric distribution and p-value < 0.05 was considered to be significant enrichment. We applied a correction of hypergeometric p-value by means of multiple hypothesis testing according to Benjamini and Hochberg [43].

### 1.7 Hub genes identification

For the hub gene identification, the genes belonging to the communities found in the step 3 were analyzed using Kleinberg’s centrality score (Kcs) [44]. The score of the vertices are defined as the principal eigenvector of *CC*^*T*^ where *C* is the adjacency matrix. In this work we considered hub of a community genes with a *Kcs* > 0.8 (the maximum value of Kcs is 1). To confirm the robustness of this selection criterion we implemented a bootstrap procedure [45, 46]. The data sample with 31 subjects was resampled 100 times and for each re-sampling we repeated the step 2 of the pipeline. For each resampling we computed the overlap between the found communities and we verified if hub genes of a given community were clusterized together. In this way we also verified the existence of a pivotal and robust module of genes more connected to AD. To quantify the overlap between two different communities we estimated the overlap coefficient *C*_*O*_ [47]. It is defined as the maximum intersection between the target community and communities obtained by a different process (or a different dataset) divided by the smaller of the size of the two sets:

$$\begin{array}{c} C_{O}\left(Q_{t},Q_{i}\right)=\frac{max(Q_{t}\cap Q_{i})}{min(|Q_{t}|,|Q_{i}|)} \end{array}$$ (6)

where *Q*_*t*_ is the target community; *i* is an index between 1 and the number of communities obtained by a different process and *Q*_*i*_ is the related community.

### 1.8 Identification of gene communities through WGCNA algorithm

We applied WGCNA algorithm to the co-expressed gene network built in the step 2 of the pipeline and compared the outcome with results obtained by means of our procedure. At first, in order to assess the similarity of the gene expression profiles, the Pearson’s correlation coefficient was calculated through Eq 2. Hence, the adjacency matrix *C* was obtained by applying a soft thresholding procedure [17]:

$$\begin{array}{c} c_{ij}=power\left(d_{ij},\beta \right)={\left(d_{ij}\right)}^{\beta } \end{array}$$ (7)

where *β* ≥ 1.

Zhang B and Horvath S. in [20] also proposed an another type of soft adjacency function, the sigmoid function:

$$\begin{array}{c} c_{ij}=sigmoid\left(d_{ij},\alpha ,\mu \right)=\frac{1}{1+e^{-\alpha (d_{ij}-\mu )}} \end{array}$$ (8)

where *α* and *μ* are parameters to set. WGCNA uses hierarchical clustering to identify gene communities and their respective colors. In this work, different communities were detected using dynamic tree cut method, which is based on conversion of the adjacency matrix to a topology overlay matrix (TOM) and cluster analysis. Finally, we computed the correlation of the module eigengene of each module detected by WGCNA with AD clinical features by means of the procedure described in the section 1.5.

### 1.9 Identification of gene communities through traditional clustering methods

We compared results obtained though our procedure with findings of traditional clustering strategies as: agglomerative hierarchical clustering, Fuzzy c-means, and affinity propagation. The common property of clustering algorithms is that they distribute genes in groups based on similarity measures in gene expression [48]. We can classify the three proposed approaches according to the way used to determine the number of modules: explicit methods, such as agglomerative hierarchical clustering and fuzzy c-means, in which the number of clusters is imposed by the researcher; implicit methods, such as affinity propagation, where the number of modules is adapted on the dataset analyzed according to other information suggested by the researcher [48]. The agglomerative hierarchical clustering method merges iteratively clusters together if their similarity measure is sufficiently high [30]. This similarity is based on a specific metric that measures the distance between pairs of elements and a linkage criterion that computes how similar clusters are according to the chosen metric. In this work we implemented the Euclidean distance (*L*_2_ − *norm*):

$$\begin{array}{c} d_{E-AB}=\sum_{j=1}^{n}\sqrt{{\left(a_{j}-b_{j}\right)}^{2}} \end{array}$$ (9)

and the Manhattan distance (*L*_1_ − *norm*):

$$\begin{array}{c} d_{M-AB}=\sum_{j=1}^{n}\left|a_{j}-b_{j}\right| \end{array}$$ (10)

where *a*_*j*_ and *b*_*j*_ are elements of two data points respectively *A* = (*a*_1_, *a*_2_, …, *a*_*n*_) and *B* = (*b*_1_, *b*_2_, …, *b*_*n*_).

We used the following linkage criteria:

- complete linkage: the maximum distance between clusters is calculated before merging;
- single linkage: the minimum distance between clusters is computed before merging;
- average linkage: the average distance between clusters is calculated before merging;
- centroid linkage: after finding centroids of clusters, the distance between them is calculated before merging.

To choose the metric and the optimal number of clusters we computed the silhouette coefficient. This coefficient quantifies how well an observation (in this work an observation is represented by a gene with a gene expression value for each considered subject) is clustered measuring the proximity of each point in a cluster to points in neighboring clusters. It’s defined as [49]:

$$\begin{array}{c} s\left(i\right)=\frac{z(i)-y(i)}{max(y(i),z(i))} \end{array}$$ (11)

in which *y*(*i*) is the average dissimilarity between *i*-*th* element and all other elements of the cluster to which belongs and *z*(*i*) is the minimum average dissimilarity of *i*-*th* element to all observation of all other clusters D. The silhouette coefficient is a quantity between −1 and 1, where a value near 1 indicates that the element *i* is very well clustered whereas a value near -1 indicates that the point should be affected to another cluster.

The Fuzzy c-means clustering algorithm (FCM) operates by randomly assigning a degree of membership *μ*_*ij*_ to each element *x*_*i*_ with belongs to each cluster *j* that we want to derive [50]. Through an iterative process, the cluster centers *c*_*j*_ are dynamically moved towards the optimal localization that is going to minimize a objective function that represents the sum of the distances of each point from each cluster center, appropriately weighted with the correct degree of membership. This function is defined as [50]:

$$\begin{array}{c} J=\sum_{i=1}^{D}\sum_{j=1}^{N}\mu_{ij}^{m}{∥x_{i}-c_{j}∥}^{2} \end{array}$$ (12)

where *μ*_*ij*_ is the degree to which an observation *x*_*i*_ belongs to a cluster *j*; *c*_*j*_ is the center of the cluster *J*; *D* is the number of observation; *N* is the desired number of clusters; *m* is the hyper-parameter (fuzzifier) that controls how fuzzy the cluster will be. In general, the squared Euclidean distance metric is used with FCM in order to compute the distances between the cluster centers and each observation in the dataset. In this work we compared the performance of three different metrics: euclidean, Manhattan, Pearson correlation defined in Eqs (9), (10) and (2) respectively.

Affinity Propagation (AF) [51] is a cluster technique that does not require user to specify the number of clusters. This algorithm is based on the concept of message passing where all the data points send messages to all other points. These messages contains the willingness of the points being exemplars i.e. the points that best explain the other data points. Each cluster only has one exemplar. The messages according to their nature are inserted in two different matrices: the responsibility matrix *R* in which each element reflects how suitable a point *k* is to be an exemplar for a point *i*; the availability matrix *A* that quantify how appropriate it would be for a point *i* to choose point *k* as its exemplar. The values of matrix *R* are based on a similarity function. The standard similarity measure used in the papers of Frey and Dueck is the negative euclidian distance squared [51]. AF is an iterative algorithm in which iterations are performed until either the cluster boundaries remain unchanged over a number of iterations. The exemplars are the points that at the last iteration satisfy the following condition:

$$\begin{array}{c} R(i,i)+A(i,i)>0 \end{array}$$ (13)

### 1.10 Identification of gene communities through three other network reconstruction algorithms

We compared our findings with other three methods for constructing gene network: ARACNE, GENIE3, and SPACE. These methods can in some cases compete with clustering and decomposition algorithms. Here we give a brief summary of these techniques. The detailed methodology for each approach has been described in other papers [52–54]. Tools such as ARACNE and GENIE3 try to build regulatory networks from co-expression networks [55]. ARACNE (Algorithm for the Reconstruction of Accurate Cellular Networks) eliminates indirect connections between genes, then partners of a gene having a stronger correlation with each other genes than with the gene itself [55]. Only those connections that should be regulatory are left. This tool is an information-theory-based method that uses mutual information instead of Pearson correlation with the advantage to identify the non-linear or irregular dependencies, which will be missed by Pearson correlation [56].

GENIE3 (GEne Network Inference with Ensemble of trees) is a network inference method based on variable selection with ensembles of regression trees [53]. This tool is able to divide the problem of prediction of a regulatory network between *n* genes into *n* different regression issues. In each of the regression problems, the expression pattern of the target gene is predicted from the expression patterns of all the other genes (input genes), by means of tree-based ensemble methods (for example Random Forests). A sign on a possible regulatory link is derived from the importance of an input gene in predicting a target gene. These possible links are then jointed over all genes to provide a ranking of interactions to build whole network [53].

SPACE (Sparse PArtial Correlation Estimation) [54] is a partial-correlation-based method. This technique points to estimate non-zero entries in the inverse of the covariance matrix, also known as the concentration matrix. In the SPACE algorithm the concentration matrix estimation problem is converted in a regression problem and the results are optimized with a symmetric constraint [56].

To compare the different proposed methods with our procedure, we computed the correlation of the module eigengene of each found communities with AD clinical features, as described in the section 1.5.

## 2 Results and discussion

### 2.1 Preprocessing procedure

**Fig 3:**
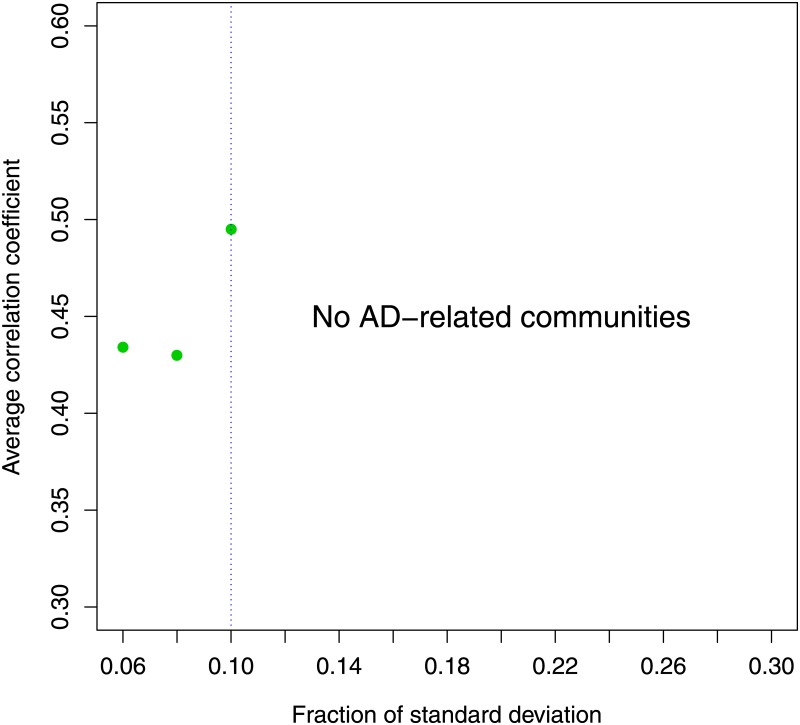
Average correlation coefficient with different cut. 0.1 *σ*_*max*_ is the optimal cut that maximizes the average correlation between the communities of interest and clinical features MMSE and NFT. In fact selecting genes exceeding 0.1 *σ*_*max*_ we did not find AD-related communities.

Firstly, we calculated the standard deviation *σ* for each gene expression, then we determined the maximum value *σ*_*max*_. In this analysis we considered only genes with standard deviation greater than 0.1 *σ*_*max*_. We chose this selection criterion after a study to evaluate the optimal cut. We repeated steps 1-3 of the pipeline for different values. 0.1 *σ*_*max*_ is resulted the optimal cut that maximizes the average correlation between the communities of interest and clinical features MMSE and NFT, as shown in Fig 3. Furthermore selecting genes exceeding 0.1 *σ*_*max*_ would have resulted in no AD-related communities (step 3 of the pipeline).

**Fig 4:**
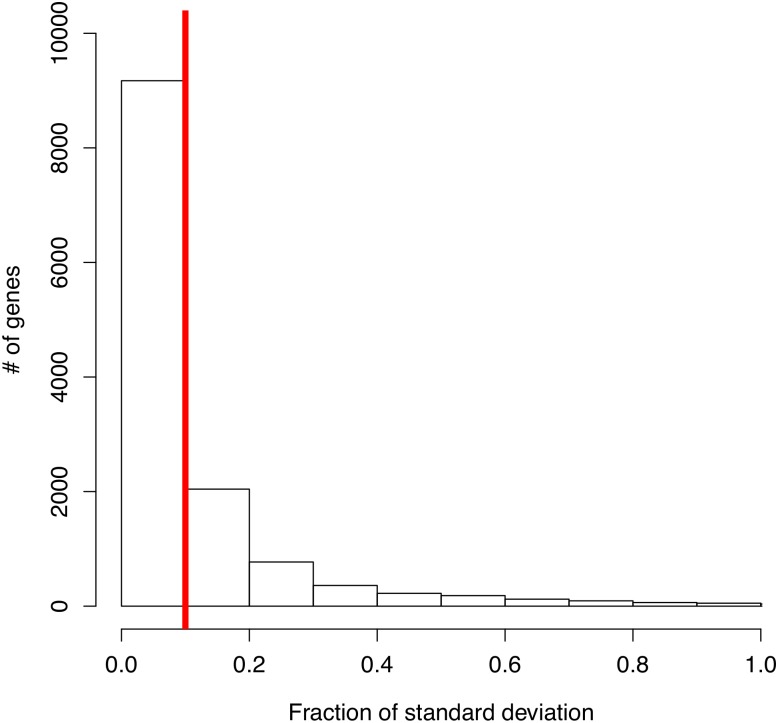
Distribution of standard deviation of gene expression. The vertical red line indicates the selection implemented on the data. Only the genes to right of the red line for further analysis have been selected.

Fig 4 illustrates the distribution of standard deviation of gene expression. Then we kept 4, 154 genes for further analysis.

### 2.2 Information entropy based on betweenness to select the best threshold value

**Fig 5:**
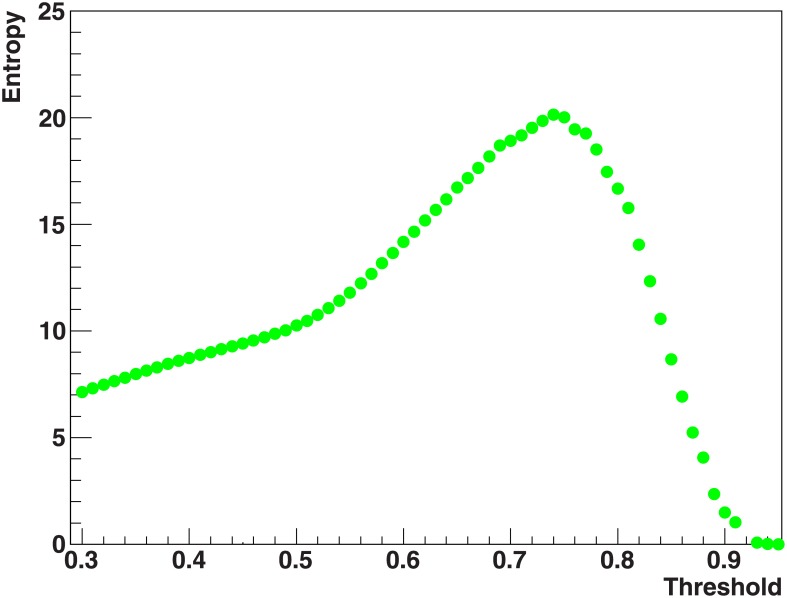
Betweenness entropy as a function of threshold. Information entropy distribution based on betweenness as a function of threshold for the whole network. The distribution presents a maximum at a threshold value equal to 0.74.

With gene expressions selected in the preprocessing procedure, we built the network of co-expressed genes. Fig 5 shows entropy distribution based on betweenness, as a function of the threshold for the co-expression network. The distribution presents a maximum at the threshold value 0.74 which corresponds to the network configuration with the highest informative significance related to betweenness.

### 2.3 Community detection through Fast Greedy algorithm and correlation analysis with clinical phenotype

We applied the Fast Greedy community detection algorithm on the network at threshold equal to 0.74 resulting 127 gene communities. The Pearson’s correlation coefficient between the principal component of communities and clinical phenotype was computed to identify communities significantly correlated with clinical features MMSE score and NFT value simultaneously. We found only two gene communities significantly associated with both clinical features:

- a community that included 72 genes (called *C*_1_);
- a community that included 39 genes (called *C*_2_).

**Fig 6:**
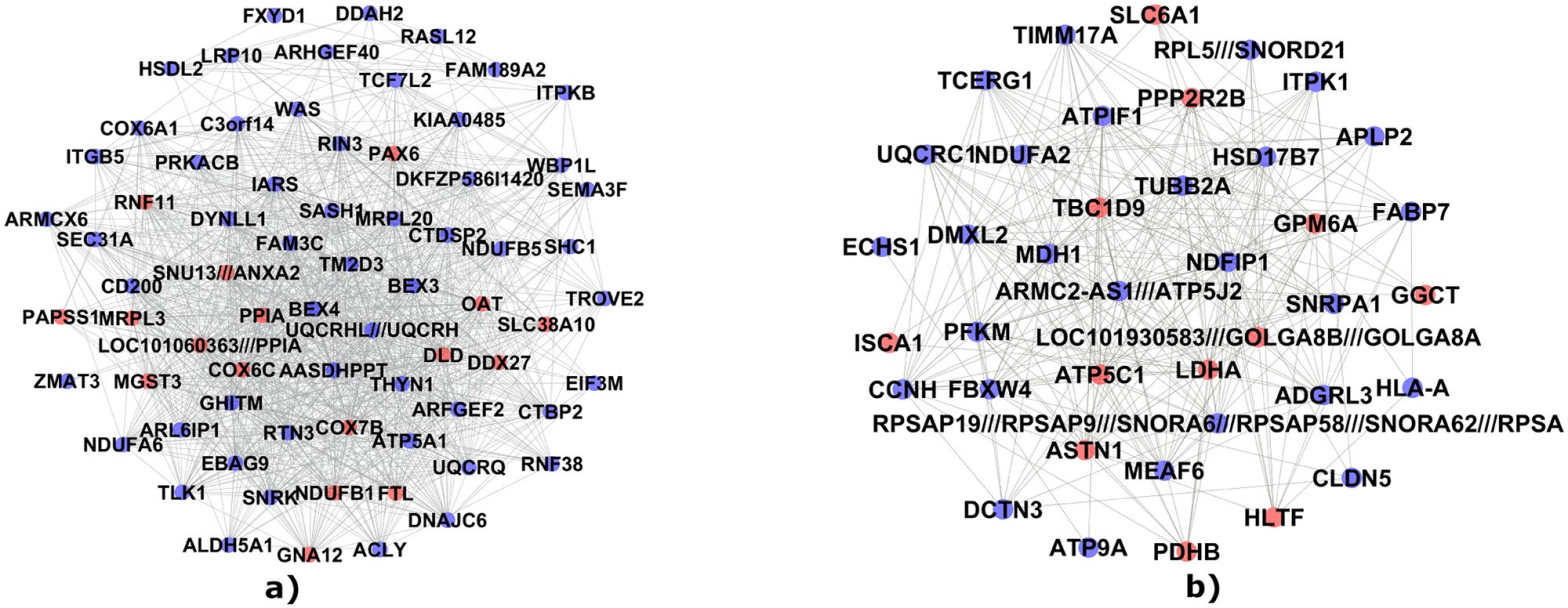
Schematization of *C*_1_ and *C*_2_ communities. Gene community *C*_1_ (Panel a) and gene community *C*_2_ (Panel b) composed by 72 and 39 genes respectively. In red we indicated genes significantly correlated with both clinical features simultaneously.

Table 1 highlights the results obtained for *C*_1_ and *C*_2_ communities from the correlation test with MMSE and NFT. Fig 6 shows a schematization of *C*_1_ and *C*_2_ communities.

**Table 1:** Results of correlation test between the first principal component of *C*_1_ and *C*_2_ communities and clinical features. The results show significant correlation (*p*-*value* < 0.05) of both gene communities with MMSE and NFT.

Community	MMSE	NFT
*C*_1_	r = -0.46; p = 0.03	r = 0.59; p = 0.004
*C*_2_	r = 0.47; p = 0.02	r = -0.46; p = 0.03

**Fig 7:**
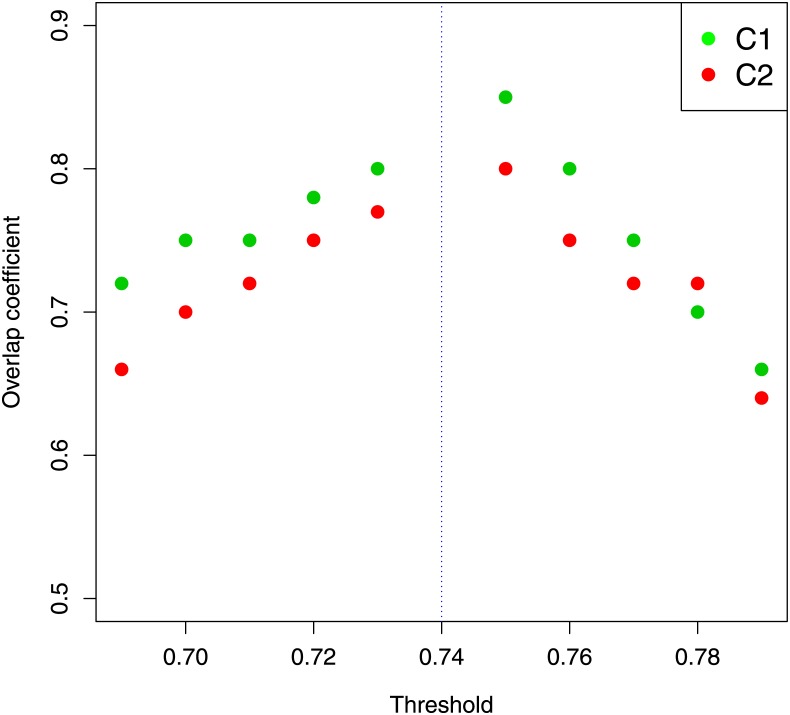
Overlap coefficient for *C*_1_ and *C*_2_ communities. The overlap coefficient as a function of threshold calculated in relation to the *C*_1_ and *C*_2_ communities. The dashed vertical line indicates the threshold value selected for our analysis.

**Fig 8:**
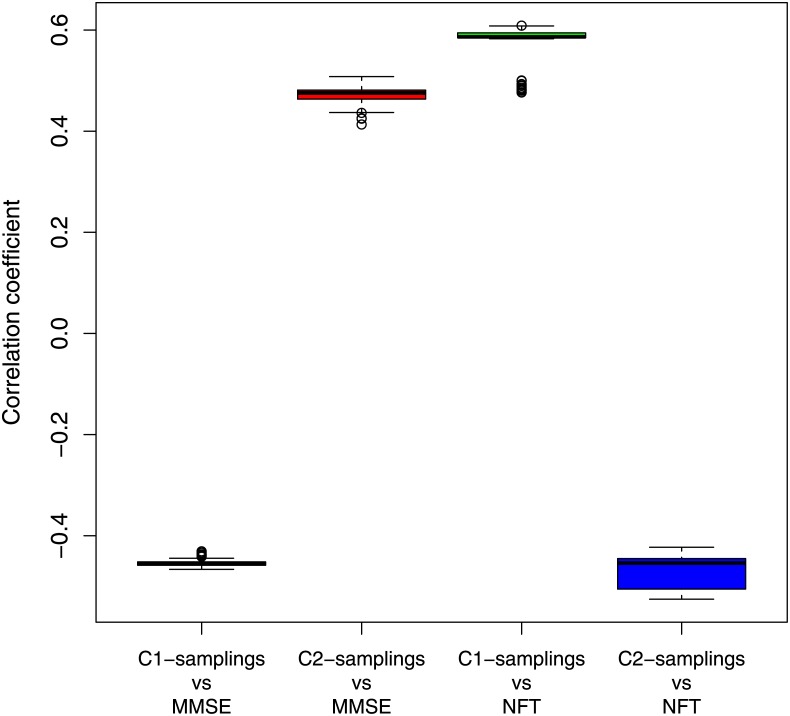
Distributions of correlation coefficient with the clinical features for 100 sampling of *C*_1_ and *C*_2_ communities. Random subsets of 10 genes are removed from *C*_1_ and *C*_2_ communities before to compute the first principal component.

We computed the overlap coefficient through Eq 6 for different threshold values belonging to neighborhood 0.74 to evaluate the stability of *C*_1_ and *C*_2_ communities. Fig 7 displays the overlap coefficient computed in relation to *C*_1_ and *C*_2_ communities for different threshold values. The high values of overlap coefficient (> 0.6) certify the stability of the two communities over a wide range of the chosen threshold. To verify the robustness of results reported in Table 1 we applied the sampling procedure described in the section 1.5 on *C*_1_ and *C*_2_ communities and obtained distributions of correlation coefficients as shown in Fig 8. Distributions appear consistent with correlation coefficient values reported in Table 1.

The gene membership per community are reported in S4 Table. In S5 Table we listed genes of *C*_1_ and *C*_2_ communities with the weight in their contribution to the eigengene and their individual correlation with MMSE and NFT variables. Finally the list of detected communities with their correlation with clinical features is given in S6 Table.

### 2.4 Gene set enrichment analysis

*C*_1_ and *C*_2_ communities have been subjected to gene set enrichment analysis. The results for *C*_1_ and *C*_2_ communities are shown in Tables 2 and 3 respectively.

**Table 2:** Results of gene set enrichment analysis for *C*_1_ community. In the third column overlaps with gene set in the selected MSigDB gene set collection are reported. The fourth column displays the false discovery rate (FDR) analog of hypergeometric p-value after correction for multiple hypothesis testing according to Benjamini and Hochberg [43]. The table shows seven most significant enrichments.

Gene Set Name	Description	Genes in overlap	FDR p-value
BLALOCK ALZHEIMER’S DISEASE DN	Genes down-regulated in brain from patients with Alzheimer’s disease—Homo sapiens	32	1.25 ⋅ 10^−28^
HALLMARK OXIDATIVE PHOSPHORYLATION	Genes encoding proteins involved in oxidative phosphorylation—Homo sapiens	11	3.87 ⋅ 10^−11^
GO ELECTRON TRANSPORT CHAIN	A process in which a series of electron carriers operate together to transfer electrons from donors to any of several different terminal electron acceptors to generate a transmembrane electrochemical gradient—Homo sapiens	9	5.24 ⋅ 10^−11^
GO CELLULAR RESPIRATION	The enzymatic release of energy from inorganic and organic compounds (especially carbohydrates and fats) which either requires oxygen (aerobic respiration) or does not (anaerobic respiration)—Homo sapiens	9	1.24 ⋅ 10^−9^
GO ORGANONITROGEN COMPOUND METABOLIC P C PROCESS	The chemical reactions and pathways involving organonitrogen compound—Homo sapiens	19	6.23 ⋅ 10^−9^
KEGG PARKINSONS DISEASE	Parkinson’s disease—Homo sapiens	19	1.38 ⋅ 10^−8^
BLALOCK ALZHEIMER’S DISEASE UP	Genes up-regulated in brain from patients with Alzheimer’s disease—Homo sapiens	18	1.38 ⋅ 10^−8^

**Table 3:** Results of gene set enrichment analysis for *C*_2_ community. In the third column the genes in the overlapping gene sets are reported. The fourth column indicates the false discovery rate (FDR) analog of hypergeometric p-value after correction for multiple hypothesis testing according to Benjamini and Hochberg [43]. he table shows the top three significant enrichments.

Gene Set Name	Description	Genes in overlap	FDR p-value
BLALOCK ALZHEIMER’S DISEASE DN	Genes down-regulated in brain from patients with Alzheimer’s disease—Homo sapiens	22	1.07 ⋅ 10^−22^
HALLMARK OXIDATIVE PHOSPHORYLATION	Genes encoding proteins involved in oxidative phosphorylation—Homo sapiens	9	1.23 ⋅ 10^−10^
KIM BIPOLAR DISORDER OLIGODENDROCYTE DENSITY CORR UP	Genes whose expression significantly and positively correlated with oligodendrocyte density in layer VI of BA9 brain region in patients with bipolar disorder—Homo sapiens	11	5.49 ⋅ 10^−9^

### 2.5 Hub genes identification

**Fig 9:**
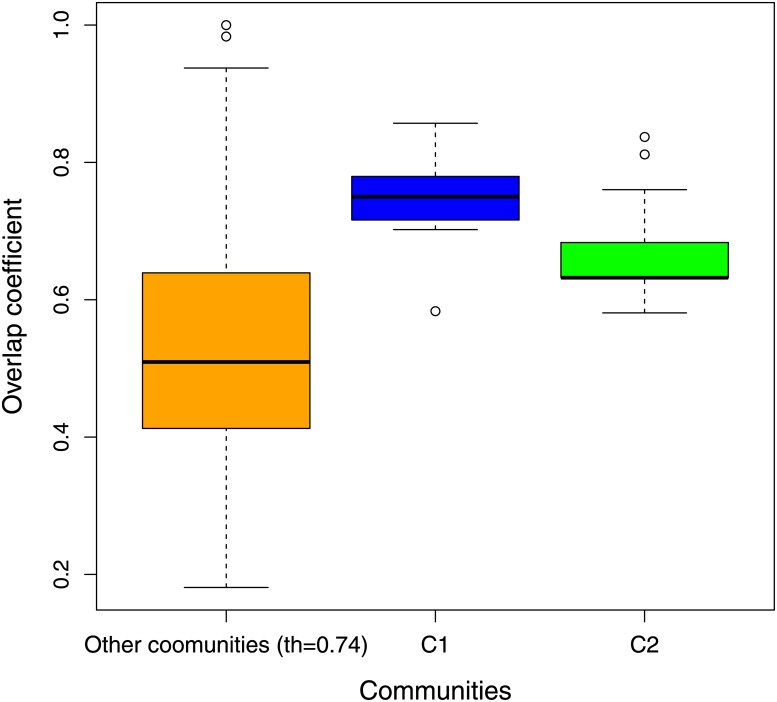
Overlap coefficient distributions for *C*_1_ and *C*_2_ communities and other communities found with *th* = 0.74. The overlap coefficient is computed for each bootstrap resampling.

We identified 24 hub genes in *C*_1_ community and 9 in *C*_2_ community with *Kcs* > 0.8. Moreover we implemented a boostrap procedure, with 100 resamplings, to confirm these hub genes. In all resamplings, 28 genes belonging to *C*_1_ community were clustered together as well as 13 of *C*_2_ community. We called these modules sub-*C*_1_ and sub-*C*_2_. Hub genes of *C*_1_ and *C*_2_ communities are included in sub-*C*_1_ and sub-*C*_2_ modules, respectively. Hence, we implemented the correlation analysis described in section 1.5 for the hub genes of *C*_1_ and *C*_2_. The findings for MMSE score ($r_{hub-C_{1}}=-0.55$, $p_{hub-C_{1}}=0.008$; $r_{hub-C_{2}}=0.60$, $p_{hub-C_{2}}=0.003$) and NFT value ($r_{hub-C_{1}}=0.55$, $p_{hub-C_{1}}=0.008$; $r_{hub-C_{2}}=-0.47$, $p_{hub-C_{2}}=0.02$) show that the correlations between these genes and clinical features are improved compared to results reported in Table 1. Lists of hub genes for *C*_1_ and *C*_2_ communities are reported in S1 Table. Fig 9 displays overlap coefficient distributions for *C*_1_ and *C*_2_ communities and other communities found with *th* = 0.74 for each bootstrap resampling. *C*_1_ and *C*_2_ communities appear to be better preserved than other communities.

### 2.6 Identification of gene communities through WGCNA

**Fig 10:**
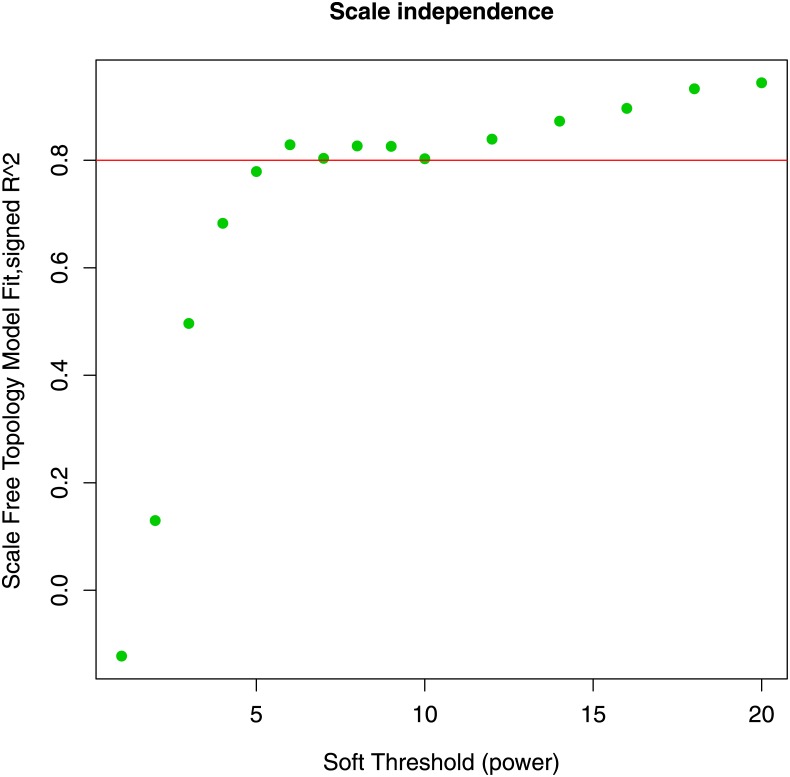
Scale free topology fitting index for different soft thresholds. *β* value equal to 6 was the smallest threshold that presented *R*^2^ greater than 0.8.

We applied WGCNA algorithm to gene expressions selected in the section 2.1. We implemented the scale independence analysis and we found *β* value equal to 6 was the smallest threshold that resulted in a scale-free *R*^2^ fit greater than 0.8, as shown in Fig 10.

**Fig 11:**
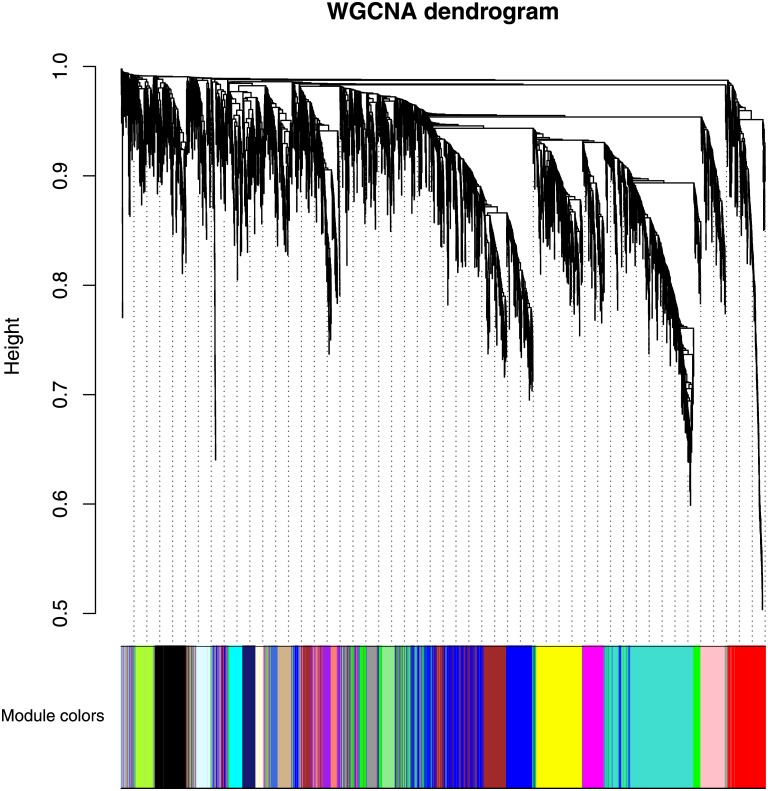
Dendrogram of the network with the modules indicated through the colors. This dendrogram was obtained through average linkage hierarchical clustering. The color spectrum underneath the plot indicates the module assignment determined by means of the Dynamic Tree Cut.

**Fig 12:**
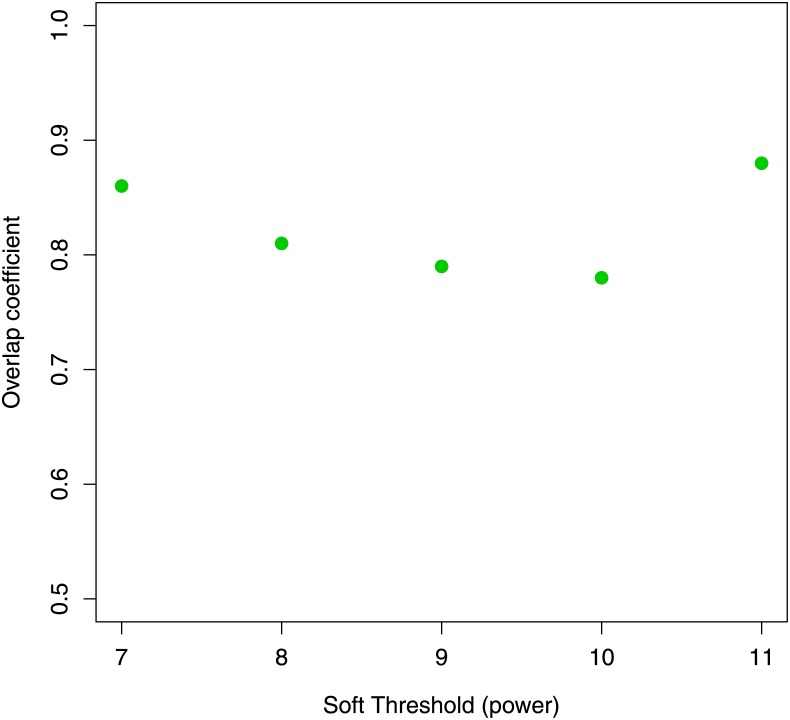
The overlap index between the blue module (*β* = 6) and other AD-related modules obtained for different threshold values. We obtained *C*_0_ > 0.75 for all different threshold values.

Through this configuration we obtained 21 gene communities. Fig 11 shows a dendrogram of 21 modules found by means of the WGCNA algorithm. We computed the linear correlation between communities and clinical features MMSE score and NFT value. Only one community, the blue module, was significantly associated with both clinical features (*r* = −0.43 with MMSE; *r* = 0.43 with NFT). This community was composed by 637 genes, and it contained the 78% of *C*_1_ and *C*_2_ communities (87 genes). All hub genes found in the previous section were included in the blue module. Fig 12 shows the overlap index between the blue module computed with *β* = 6 and other AD-related modules obtained for different threshold values. A strong module overlap emerges for different threshold values (*C*_*O*_ > 0.75) confirming the goodness of the choosen *β*. In S7 Table we reported for each threshold values and for different parameter values of sigmoid function the number of modules detected and their correlation with the clinical features. Topological measures of co-expression network constructed through WGCNA and our method are reported in S2 and S3 Tables.

### 2.7 Identification of gene communities through traditional clustering methods and network reconstruction algorithms

**Fig 13:**
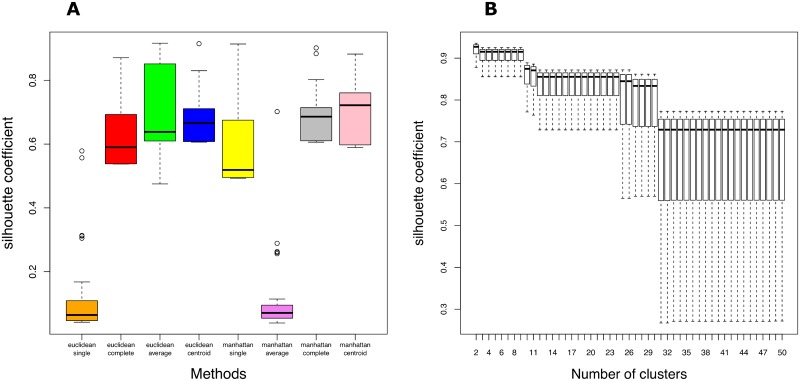
Silhouette coefficient distributions for agglomerative hierarchical clustering method. Silhouette coefficient distributions computed for different configurations (Panel A), and for different number of clusters (Panel B) only for the chosen configuration Manhattan-centroid.

On gene expressions selected in the section 2.1 we implemented other seven methods of community detection. Fig 13 displays the silhouette coefficient obtained by means of Eq 11 for agglomerative hierarchical clustering in many configurations. In particular in the panel A we presented distributions of the silhouette coefficient for the metrics and linkage criteria listed in the section 1.9. For each proposed methods we considered the number of clusters between 2 and 50. Instead Panel B shows the silhouette coefficient as a function of the number of clusters *k* for the chosen configuration (Manhattan-centroid). Based on the distribution of the silhouette coefficient, we chose *k* between 2 and 30 and we found no AD-related modules.

**Fig 14:**
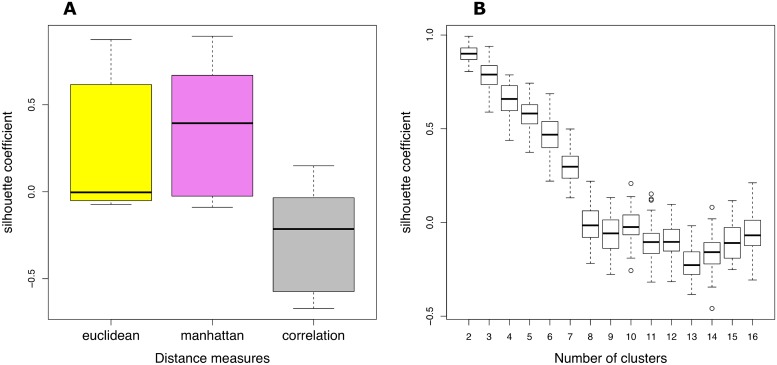
Silhouette coefficient distributions for fuzzy c-means method. Silhouette coefficient distributions computed for different distance measures (Panel A), and for different number of clusters (Panel B) only for the chosen metric Manhattan.

In Fig 14 we reported silhouette coefficient distributions computed for the Fuzzy c-means, for three different distance metrics and for a number of clusters between 2 and 50 (Panel A). Pick out Manhattan as metric, in panel B we reported the silhouette coefficient for a set of clusters number. Choosing *k* between 2 and 7 (since the average silhouette coefficient is greater than 0) no module was correlated with AD. In this work the default parameters were used for affinity propagation, ARACNE, GENIE3, and SPACE without additional tuning. Table 4 highlights the results obtained through the selected community detection methods.

**Table 4:** Summary table of the results obtained through the proposed community detection methods. In the fourth column the number of genes in common with *C*_1_ and *C*_2_ communities is shown in bold. We reported only significant correlations (*p*-*value* < 0.05).

Method	Number of communities	AD-related communities	Number of genes in AD-related communities	Correlation coefficient with NFT	Correlation coefficient with MMSE
Our method	127	*C*_1_	71	0.59	−0.46
		*C*_2_	39	−0.46	0.47
WGCNA	21	Blue module	637(**87)**	0.43	−0.43
Affinity propagation	233	*A*_1_	31(**7)**	0.45	−0.43
		*A*_2_	39(**23)**	0.45	−0.43
Agglomerative clustering	Between 2 and 30	None			
Fuzzy c-means	Between 2 and 7	None			
ARACNE	674	*AR*_1_	8(**2)**	−0.45	0.44
		*AR*_2_	7(**5)**	−0.43	0.43
GENIE3	675	*G*_1_	9(**3)**	−0.44	0.44
		*G*_2_	7(**2)**	−0.44	0.46
SPACE	24	*S*_1_	25(**2)**	0.44	−0.44

### 2.8 Validation on independent datasets

**Fig 15:**
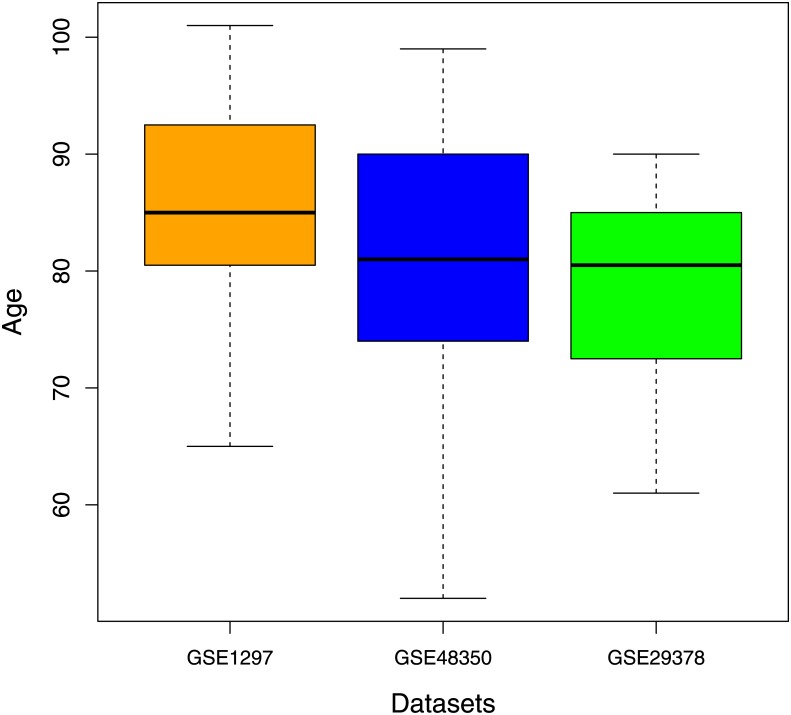
Age distributions of the three datasets analyzed. To make the three distributions comparable we did not consider 17 samples of young subjects in the database GSE48350.

Due to the relationship between aging and AD [57] we excluded the gene expression profiles of young (20-50 years) from GSE48350. In this way, we made comparable the three dataset age distributions, as displayed in Fig 15.

For GSE48350 database we found a first community composed by 35 genes with overlap of 23 with *C*_1_ community (8 hub genes of *C*_1_) and a second community of 28 genes, 23 members of *C*_2_ community (8 hub genes of *C*_2_). Instead applying our procedure on GSE29378 database we obtained a first community with 79 genes, 49 members of *C*_1_ community (17 hub genes of *C*_1_) and a second community containing 35 genes with overlap of 21 genes with *C*_2_ community (5 hub genes of *C*_2_). These findings highlight the replicability of our results on unseen datasets. Results of gene set enrichment analysis of these communities found for GSE48350 and GSE29378 are reported in S8 Table.

### 2.9 Discussion

In the present study, we implemented a co-expression network-based approach to analyze the whole genome expression data obtained from AD and control human hippocampus. The selected database GSE1297 was accompanied by the complete information of gene expression data and detailed clinical data. We applied a hard threshold analysis, proposed in a previous work [21], in which we analyzed the Shannon entropy based on betweenness of the network. We selected the network configuration with the highest informative significance. Through this procedure we identified 127 gene communities. Due to the high threshold value selected (*th* = 0.74), genes in each community showed highly-related expression, indicating potential interaction and a common biological trend. By calculating the Pearson’s correlation coefficient between the communities and two AD-related clinical features such as MMSE score and NFT value, we selected two communities (called *C*_1_ and *C*_2_) of interest. *C*_1_ and *C*_2_ communities were composed by 72 and 39 genes respectively and they showed significant but opposite correlations with the same variables (see Table 1). We compared our method with three traditional clustering algorithms: agglomerative hierarchical clustering, Fuzzy c-means, affinity propagation and with four network reconstruction approaches: WGCNA ARACNE, GENIE3, and SPACE. As shown in Table 4 these other methods obtained communities with slightly lower correlations with clinical variables than *C*_1_ and *C*_2_ communities. In particular through WGCNA algorithm we found only one community of interest: the blue module. This community was quite large (637 genes) and the 78% of *C*_1_ and *C*_2_ communities was included in it. Our method preserved and condensed into two much smaller communities the relevant information highlighted by WGCNA. Furthermore, our analysis confirmed a limitation of the WGCNA approach, in which small gene communities may, in some cases, be incorporated into larger modules, with a loss of biological information [17]. Instead ARACNE and GENEIE3 algorithms suppress too many connections by creating a very large number of small communities. Compared to the remaining implemented techniques our findings are similar in terms of correlation to AD but *C*_1_ and *C*_2_ communities hold a different information content because they consist largely of different genes. The proposed approach outlines the relationships among genes and paves the way to further studies about the physiological interpretation of AD-related communities. To assess the reproducibility of our findings, we performed our community detection method on two independent datasets: GSE48350 and GSE29378. We obtained two communities for each datasets within at least the 65% of *C*_1_ and *C*_2_ genes. These results underline the importance of the found communities in relation to AD. In previous work a lot of genes belonging to *C*_1_ and *C*_2_ communities had already been highlighted as linked to AD. For example RIN3 was found to play key roles in the development of AD ad in particular it resulted associated with AD risk [58, 59]. J.W. Liang et al. [40] reported an AD-related community containing RIN3 and GNA12, two genes belonging to *C*_1_. Seyfried N.T. et al. [60] by means of a co-expression network analysis found 10 modules correlated with AD phenotype. In particular in M1 module (composed by 1294 genes) were contained 12 genes found in *C*_1_ (PPIA, RTN3, COX6C, DNAJC6, AASDHPPT, COX7B, TM2D3, RNF11, BEX4, CD200, MRPL3, THYN1) and 9 found in *C*_2_ (TUBB2A, GPM6A, MDH1, PFKM, ATP9A, UQCRC1, ASTN1, ATPIF1, GGCT) while in M5 module (composed by 775 genes) were clustered together 9 genes of *C*_1_ (PRKACB, OAT, GHITM, PAPSS1, IARS, NDUFA6, ACLY, TLK1, C3orf14) and 4 of *C*_2_ (LDHA, PDHB, ISCA1, TCERG1). Miller J.A. et al. [61] used WGCNA to identify 12 distinct modules related to synapticand metabolic processes of AD. Specifically we found a good overlap with the module enriched in mitochondrion pathway (containing 366 genes): 13 genes belonging to *C*_1_ community (COX6C, ATP5A1, OAT, AASDHPPT, GHITM, COX7B, TM2D3, IARS, FAM3C, NDUFB5, DLD, CD200, MRPL3) and 9 to *C*_2_ community (MDH1, ATP5C1, NDFIP1, PDHB, PFKM, UQCRC1, TIMM17A, CCNH, ATPIF1).

Further gene set enrichment analysis showed that both communities *C*_1_ and *C*_2_ were mostly enriched in AD, oxidative phosphorylation (OXPHOS), Krebs (TCA) cycle, Parkinson’s disease and bipolar disorder pathways. Several works studied the pathway enrichments associated with AD [62–64]. For example, Naj et al. [65] provided a comprehensive review of genomic studies of AD. Moreover, many studies [66, 67] confirmed that oxidative phosphorylation and electron transfer defects were closely associated with neuro-degenerative diseases, such as AD. Oxidative phosphorylation rapresents the apex of a series of energy transformations indicated as cellular respiration or simply respiration in their entirety [68]. In this cellular process the electron transport chain constitutes a proton gradient across the inner mitochondrial membrane, in which the synthesis of ATP is driven through the chemiosmosis. The role of OXPHOS changes in the pathogenesis of AD is controversial. Abnormalities in cellular bioenergetics have been detected in a lot of people affected by AD and their links with dementia have been highlighted in several experiments in vivo and in vitro [69]. As reported in Table 2, *C*_1_ community results also enriched for genes involved in Krebs (TCA) cycle. The TCA cycle is a mitochondrial metabolic process essential for generating the proton gradient across the inner membrane of the mitochondria that is used to produce ATP [70]. A connection between aberrations in TCA cycle and AD has been widely witnessed [70–73]. For example, Bubber et al. [74] stated that the metabolic activity of the TCA cycle decreases in AD mitochondria due to a decline in several of the enzymes of the cycle. As reported in S8 Table also the found communities within GSE48350 and GSE29378 were mostly enriched in AD, OXPHOS and energy cellular processes pathways. Finally, we implemented a study of hub genes by means of the Kleinberg’s centrality and identified 24 hub genes in *C*_1_ community and 9 in *C*_2_ community. The robustness of our findings was confirmed through a bootstrap procedure. In fact, *C*_1_ and *C*2 communities appear on average more stable than the other communities, as displayed in Fig 9. The communities of the hub genes presented a stronger correlation with the clinical phenotype than *C*_1_ and *C*_2_ communities. This could indicate the presence of a robust core component of genes within the detected communities more closely related to AD. As shown in Fig 6 and reported in S5 Table the found hub nodes often do not coincide with the genes most correlated to AD phenotype. In fact our procedure was based on a centrality measure and a bootstrap verification independently by correlation with clinical features. The detected communities, *C*_1_ and *C*_2_, and in particular the communities of their hub genes could help to understand the mechanisms of Alzheimer’s disease and they can be potential targets for a future AD therapy. Clearly, a more in-depth clinical validation is necessary to understand how these genes are implicated in the biological processes linked to AD.

## 3 Conclusions

In this paper we implemented a method proposed in a previous work and used in an international competition to detect gene communities linked to AD. Our strategy was based on a co-expression network analysis and a study of Shannon entropy of the betweenness. The pivotal role of co-expression networks consists in representing binary relationships between individual genes that may highlight obscure processes of cellular communication. As reported in Table 4 the communities found with our method are significantly smaller than the module obtained through WGCNA but much more populated than the communities detected by means of ARACNE and GENIE3 where gene connections appear suppressed. Compared to the remaining implemented techniques our results are similar in terms of correlation to AD but the two found communities have a different information content because they consist largely of different genes. These communities were stable and mostly replicated on two independent databases. Furthermore they contained some genes already known to be linked to AD as RIN3 and GNA12. The detected communities resulted mostly enriched in pathways closely associated with neuro-degenative diseases as energy cellular processes. A study of the hub genes of the two communities revealed even stronger correlation of hub genes communities to the clinical phenotype. Further exploration on the two AD-relevant communities and the detected hub genes, combined with analysis using different clinical tools such as neuroimaging [75], are needed to understand physiological mechanisms of AD.

## Supporting information

S1 Table Lists of hub genes of *C*_1_ and *C*_2_ communities. (PDF)

S2 Table Topological parameters of co-expression network constructed using WGCNA algorithm. (PDF)

S3 Table Topological parameters of co-expression network constructed using the proposed method [21]. (PDF)

S4 Table The gene membership per community found by means of the proposed method. (XLSX)

S5 Table Lists of *C*_1_ and *C*_2_ genes containing the weight in their contribution to the eigengene and their individual correlation with MMSE and NFT variables. (XLSX)

S6 Table Lists of detected communities with their correlation with clinical features. (XLSX)

S7 Table Modules detected trough WGCNA and their correlation with the clinical features in function of threshold values. (XLSX)

S8 Table Results of gene set enrichment analysis of the detected communities in GSE48350 and GSE29378 databases. (PDF)
