# Supplementary material for: Shannon entropy approach reveals relevant genes in Alzheimer’s disease
Source: PLoS One. 2019 Dec 31;14(12):e0226190. doi: 10.1371/journal.pone.0226190 (PMC6938408; doi:10.1371/journal.pone.0226190)
Supplement: S1 Table — (PDF) [file pone.0226190.s001.pdf]

S1 Table. Lists of hub genes for C_1 and C_2 communities.

C_1 community	Gene	C_2 community	Gene
	TM2D3		NDFIP1
	BEX4		ARMC2-AS1///ATP5J2
	BEX3		TBC1D9
	PPIA		LDHA
	UQCRHL///UQCRH		ATP5C1
	THYN1		TUBB2A
	AASDHPPT		MDH1
	COX6C		DMXL2
	SASH1		PFKM
	DLD		
	LOC101060363///PPIA		
	COX7B		
	RTN3		
	IARS		
	GHITM		
	CTDSP2		
	ATP5A1		
	SNU13///ANXA2		
	FAM3C		
	OAT		
	DYNLL1		
	CD200		
	MRPL20		
	MGST3		
