# Supplementary material for: Shannon entropy approach reveals relevant genes in Alzheimer’s disease
Source: PLoS One. 2019 Dec 31;14(12):e0226190. doi: 10.1371/journal.pone.0226190 (PMC6938408; doi:10.1371/journal.pone.0226190)
Supplement: S2 Table — (PDF) [file pone.0226190.s002.pdf]

S2 Table. Topological parameters of co-expression network constructed by means of WGCNA algorithm

Average cluster coefficient	0.07 ± 0.03
Average whole network connectivity	60.55 ± 7.07
Average intramodular connectivity of Blue module	27.81 ± 3.28
Average extra-modular connectivity of Blue module	49.12 ± 6.04
Average total connectivity of Blue module	76.93 ± 9.16
