# Supplementary material for: Shannon entropy approach reveals relevant genes in Alzheimer’s disease
Source: PLoS One. 2019 Dec 31;14(12):e0226190. doi: 10.1371/journal.pone.0226190 (PMC6938408; doi:10.1371/journal.pone.0226190)
Supplement: S3 Table — (PDF) [file pone.0226190.s003.pdf]

S3 Table. Topological parameters of co-expression network constructed by means of method proposed in Monaco et al. 2018.

Average cluster coefficient	0.42 ± 0.09	
Average whole network connectivity	90.21 ± 58.88	
Average intramodular degree of C1 and C2 communities	34.33 ± 12.5	14.97 ± 5.75
Average extra-modular degree of C1 and C2 communities	220.10 ± 110.50	193.10 ± 102.75
Average total degree of C1 and C2 communities	254.43 ± 117.50	208.08 ± 109.75
