# Supplementary material for: Shannon entropy approach reveals relevant genes in Alzheimer’s disease
Source: PLoS One. 2019 Dec 31;14(12):e0226190. doi: 10.1371/journal.pone.0226190 (PMC6938408; doi:10.1371/journal.pone.0226190)
Supplement: S8 Table — (PDF) [file pone.0226190.s008.pdf]

S8A Table. Results of gene set enrichment analysis for the detected communities in the database GSE48350 with maximum overlap with C_1.

**Gene Set Name**	**Description**	**Genes in overlap**	**FDR p-value**
BLALOCK ALZHEIMER’S DIS- EASE DN	Genes down-regulated in brain from patients with Alzheimer’s disease - Homo sapiens	12	$1.12 \bullet 10^{-6}$
GO MITOCHONDRION	A semiautonomous self replicating organelle that occours in varying numbers, shapes and sizes in the cytoplasm of virtuality all eukaryotic cells.	9	$3.9 \bullet 10^{-3}$
GO SODIUM CHANNEL REGULATOR ACTIVITY	Modulates the activity of a sodium channel	3	$4.59 \bullet 10^{-3}$

S8B Table. Results of gene set enrichment analysis for the detected communities in the database GSE48350 with maximum overlap with C_2.

**Gene Set Name**	**Description**	**Genes in overlap**	**FDR p-value**
BLALOCK ALZHEIMER’S DIS- EASE DN	Genes down-regulated in brain from patients with Alzheimer’s disease - Homo sapiens	19	$1.31 \bullet 10^{-19}$
HALLMARK OXIDATIVE PHOS- PHORYLATION	Genes encoding proteins involved in oxidative phos- phorylation - Homo sapiens	5	$3.11 \bullet 10^{-4}$
KIM BIPOLAR DISORDER OLIGODENDROCYTE DENSITY CORR UP	Genes whose expression significantly and positively correlated with oligodendrocyte density in layer VI of BA9 brain region in patients with bipolar disorder - Homo sapiens	7	$3.11 \bullet 10^{-4}$

S8C Table. Results of gene set enrichment analysis for the detected communities in the database GSE29378 with maximum overlap with C_1.

Gene Set Name	Description	Genes in overlap	FDR p-value
BLALOCK ALZHEIMER'S DIS- EASE DN	Genes down-regulated in brain from patients with Alzheimer's disease - Homo sapiens	25	$2.61 \cdot 10^{-17}$
GO MITOCHONDRION	A semiautonomous self replicating organelle that occours in varying numbers, shapes and sizes in the cytoplasm of virtuality all eukaryotic cells.	17	$1.04 \cdot 10^{-6}$
HALLMARK OXIDATIVE PHOS- PHORYLATION	Genes encoding proteins involved in oxidative phos- phorylation - Homo sapiens	5	$3.11 \cdot 10^{-4}$

S8D Table. Results of gene set enrichment analysis for the detected communities in the database GSE29378 with maximum overlap with C_2.

Gene Set Name	Description	Genes in overlap	FDR p-value
BLALOCK ALZHEIMER'S DIS- EASE DN	Genes down-regulated in brain from patients with Alzheimer's disease - Homo sapiens	17	$9.56 \cdot 10^{-14}$
KIM BIPOLAR DISORDER OLIGODENDROCYTE DENSITY CORR UP	Genes whose expression significantly and positively correlated with oligodendrocyte density in layer VI of BA9 brain region in patients with bipolar disorder - Homo sapiens	9	$1.30 \cdot 10^{-5}$
HALLMARK OXIDATIVE PHOS- PHORYLATION	Genes encoding proteins involved in oxidative phos- phorylation - Homo sapiens	5	$1.16 \cdot 10^{-3}$
